# Postoperative outcomes in transplant patients undergoing ventral hernia repair

**DOI:** 10.1007/s00464-025-12249-4

**Published:** 2025-09-26

**Authors:** William J. Hlavinka, Kartik S. Akkihal, Kelly M. Dickerson, Daylon A. Farias, Monica Marroquin, Gerald O. Ogola, Mazen Iskandar, Marc A. Ward, Steven G. Leeds, Bola Aladegbami

**Affiliations:** 1 Division of Minimally Invasive Surgery, Baylor Scott & White University Medical Center, Dallas, TX USA; 2 Texas A&M College of Medicine, Bryan, TX USA; 3 Health Science Center, University of North Texas, Fort Worth, TX USA; 4 Baylor Scott & White Research Institute, Dallas, TX USA; 5 Department of Surgery, Roberts Hospital Department of Surgery, Baylor University Medical Center at Dallas, 3500 Gaston Ave., Dallas, TX 75246 USA; 6 Center for Advanced Surgery, Baylor Scott & White University Medical Center, Dallas, TX USA

**Keywords:** Ventral hernia, Transplant, Immunosuppression, Ventral hernia working group, Hernia

## Abstract

**Background:**

Transplant patients undergoing ventral hernia repair are classified as Ventral Hernia Working Group 2, indicating higher risk for adverse outcomes. This study assessed perioperative outcomes in transplant patients undergoing ventral hernia repair compared to a case-matched cohort of non-transplant patients.

**Methods:**

A retrospective review was conducted using a prospectively maintained database of patients who underwent ventral hernia repair between January 2018 and March 2024. Inclusion criteria included prior liver, kidney, heart, lung, or islet cell transplant patients. A 1:1 case-match was performed. Primary outcomes included 30-day complications and hernia recurrence at a mean follow-up of 13 months.

**Results:**

In total, 253 patients were included. Thirty-two transplant patients were matched to 32 patients in the control group. No difference was observed in 30-day postoperative complications including SSOs (*p* = 0.13) and SSIs (*p* = 0.16). The hernia recurrence rate was 12.1 and 3.0% in the transplant and non-transplant cohorts, respectively (*p* = 0.16).

In the transplant cohort alone, hernia recurrence was higher in patients receiving absorbable mesh compared to non-absorbable mesh (23.1% vs. 2.9%, *p* = 0.06), non-midline versus midline hernias (25.0% vs. 0.0%, *p* = 0.02), and patients on sirolimus or other immunosuppression medications versus tacrolimus (40.0% vs. 15.4% vs. 0.0%, *p* = 0.01). Two of the recurrences were bridged repairs.

**Conclusions:**

In our study we did not observe increased risk of adverse outcomes in midline hernia repairs in transplant patients. However, non-midline ventral hernias significantly impact ventral hernia outcomes in transplant patients. In a case-matched cohort, there was no increased rates of SSI or SSO in transplant patients compared to nontransplant patients, but hernia recurrence rates were higher in the transplant cohort.

Ventral hernias are an increasingly prevalent diagnosis accountable for a large population and financial burden. One in five adults experience a primary ventral hernia in their lifetime [1]. Furthermore, 30% of midline abdominal incisions result in the development of incisional hernias [1]. These hernias account for a financial burden of nearly $10 billion in the United States [2]. Additionally, serious complications after ventral hernia repair (VHR) can cost close to $27,000 in a single patient [3]. Similarly, the number of solid organ transplants continues to increase each year. As of 2024, the United Network of Organ Sharing reported a record-setting 48,149 organ transplants [4]. Since risk factors for the development of incisional hernias include obesity, immunosuppressive agents, and postoperative wound complications, this makes organ transplant recipients prime candidates for the development of incisional hernias [5, 6]. The incidence varies depending on transplant type with kidney transplants resulting in ventral hernia development in 1.1–7.0%, while liver transplant recipients have a higher rate of 9.5–32.4% [7–12].

There is a subtle gap in the literature regarding transplant patients undergoing VHR. Because of the use of immunosuppressants in transplant patients, these patients are classified as Ventral Hernia Working Group 2 (VHWG2). This matches them with other higher-risk patients with diabetes, chronic obstructive pulmonary disease, obesity, or active smoking [13]. Immunosuppression alone has been implicated in increased rates of surgical site occurrences (SSO) after open VHR but not surgical site infections (SSI). Systematic review data shows that hernia recurrence may be more likely in VHWG2 patients; however, these studies have not been limited to transplant patients [14]. Some data suggest that non-absorbable mesh is indicated in VHWG2 repairs, resulting in fewer hernia recurrences, but this is not specific to transplant patients with immune suppression [15]. A more solid foundation for mesh selection in these patients may decrease the recurrence rate. The aim of this study was to evaluate outcomes in transplant patients who underwent VHR based on mesh selection, transplant type, ventral hernia location, and immunosuppressing agent.

## Materials and methods

An institutional review board approved prospective database was retrospectively reviewed, identifying 253 patients with a ventral hernia between January 2018 and March 2024. From this database, patients were included in the transplant cohort if they were recipients of a previous liver, kidney, heart, lung, islet cell, or pancreas transplant before VHR. Basic demographics were collected such as age, gender, and race. Case-matched analysis was performed using matching data that included age, gender, race, body mass index (BMI) at the time of surgery, hypertension, diabetes mellitus, chronic obstructive pulmonary disease, current or previous smoking status, hernia size, and Center for Disease Control and Prevention (CDC) wound status.

The primary outcomes of this study included hernia recurrence and 30-day postoperative outcomes. The chief 30-day outcomes included SSO and SSI. SSO was defined as a 30-day postoperative, non-infectious complication near the incisional site and included a non-healing wound, fascial dehiscence, seroma, hematoma, and fistula. SSI was defined as a 30-day postoperative infection at the incision site and was categorized into three types: superficial (involving the skin and subcutaneous tissue), deep (involving the muscle and fascial layers), and organ/space (involving intra-abdominal structures). Additional 30-day outcomes consisted of ileus, small bowel obstruction (SBO), urinary tract infection (UTI), acute kidney injury (AKI), pulmonary embolus (PE), deep venous thrombosis (DVT), pneumonia, respiratory failure, respiratory failure requiring intubation, unplanned transfer to the intensive care unit, mortality, emergency department (ED) visit, and readmission.

Sub-set analyses were performed on the transplant cohort which assessed the above primary endpoints to evaluate the impact of transplant type, non-absorbable or absorbable mesh, hernia location (midline vs. non-midline), and immunosuppressive therapy. Absorbable mesh was defined as mesh absorbed by the body over time, while non-absorbable mesh was defined as mesh types that are permanent implants to provide long-term reinforcement. Midline and non-midline/lateral hernias were classified by the European Hernia Society classification system.

Continuous variables were presented as mean and standard deviation or medians with interquartile ranges, while categorical variables were presented as frequencies and percentages of events. Differences between continuous data were compared by the Student’s *t*-test or Kruskal–Wallis rank sum test, while Pearson’s Chi-square or Fisher’s exact tests were used to compare differences in proportion between the groups, as appropriate.

Statistical analyses were conducted with R version 4.0.3 statistical software. All statistical tests were two-sided with a statistical significance level set at p values < 0.05.

## Results

A total of 253 patients were identified across the study, with 47 patients meeting the inclusion criteria for the transplant cohort. Demographic and clinical characteristics between transplant recipients (*N* = 47) and non-transplant patients (*N* = 206) prior to matching are outlined in Table 1. The average age was similar between groups (60.3 years for transplant vs 58.4 years for non-transplant, *p* = 0.37). Body Mass Index (BMI) was similar between the groups (29.9 for transplant vs 30.9 for non-transplant, *p* = 0.34). The mean follow-up time was 13 months.

**Table 1:** Transplant vs non-transplant patient characteristics

Patient Characteristics	Transplant (N = 47)	Non-Transplant (N = 206)	*p* value
Age	60.3 (8.7)	58.4 (13.8)	0.37
Sex			0.12
Male	29 (61.7%)	101 (49.0%)	
Female	18 (38.3%)	105 (51.0%)	
Race			0.05
White	43 (91.5%)	185 (89.8%)	
Black	2 (4.3%)	20 (9.7%)	
Other	2 (4.3%)	1 (0.5%)	
Hispanic	11 (24.4%)	37 (18.0%)	0.32
BMI	29.9 (5.2)	30.9 (6.1)	0.34
Hypertension	30 (66.7%)	109 (52.9%)	0.09
Diabetes	19 (42.2%)	29 (14.1%)	< 0.01
COPD	6 (13.3%)	8 (3.9%)	0.01
Smoker			0.75
Current smoker	3 (6.5%)	10 (4.9%)	
Past smoker	12 (26.1%)	64 (31.1%)	
Never smoker	31 (67.4%)	132 (64.1%)	
CDC wound class			0.11
I	42 (89.4%)	193 (93.7%)	
II	4 (8.5%)	7 (3.4%)	
III	0 (0.0%)	1 (0.5%)	
IV	1 (2.1%)	5 (2.4%)	
Approach			0.06
Open	42 (89.4%)	162 (78.6%)	
Laparoscopic	0 (0.0%)	8 (3.9%)	
Robotic	5 (10.6%)	36 (17.5%)	
Component separation performed			< 0.01
None	24 (51.1%)	162 (78.6%)	
Transversus abdominis release	22 (46.8%)	27 (13.1%)	
External oblique release	1 (2.1%)	14 (6.8%)	
Location of mesh placement			< 0.01
None	0 (0.0%)	47 (22.8%)	
Underlay	0 (0.0%)	4 (1.9%)	
Preperitoneal	30 (63.8%)	76 (36.9%)	
Retromuscular	2 (4.3%)	8 (3.9%)	
Bridged	2 (4.3%)	1 (0.5%)	
Onlay	0 (0.0%)	3 (1.5%)	
Retrorectus	13 (27.7%)	65 (31.6%)	
Retroperitoneal	0 (0.0%)	2 (1.0%)	
Mesh fixation			< 0.01
None	39 (83.0%)	84 (40.8%)	
Sutures	8 (17.0%)	120 (58.3%)	
Tacker	0 (0.0%)	2 (1.0%)	

*BMI* body mass index; *COPD* chronic obstructive pulmonary disease; *PE* pulmonary embolism; *DVT* deep vein thrombosis

Thirty-two patients from the transplant cohort were matched to 32 patients from the non-transplant cohort. After matching analysis, no matching criteria demonstrated a significant difference, as shown in Table 2. Results of our matched analysis is shown in Table 3. No difference was found in the transplant cohort compared to the non-transplant cohort when evaluating SSOs (6.1% vs. 18.2%, *p* = 0.13) as well as SSIs (3.0% vs. 12.1%, *p* = 0.16). Ileus was seen at a rate of 12.1% in the transplant cohort compared to 3.0% in the non-transplant group (*p* = 0.30). UTI (6.1%, *p* = 0.15), pneumonia (3.0%, *p* = 0.31), and respiratory failure (6.1%, *p* = 0.15) occurred exclusively in the transplant cohort. Transplant patients were readmitted at an 18.2% rate while non-transplant patients were readmitted at a 9.1% rate (*p* = 0.17). Any 30-day complications occurred at a similar rate between the transplant and non-transplant cohorts (36.4% vs. 24.2%, *p* = 0.28). Hernia recurrences occurred at a rate of 12.1% in the transplant cohort compared to 3.0% of the non-transplant cohort (*p* = 0.16). The median time for recurrence was shorter in the transplant cohort (9.8 months vs. 14.2 months, *p* = 0.53).

**Table 2:** Matched analysis patient characteristics

Patient characteristics	Transplant (N = 33)	Non-Transplant (N = 33)
Age	59.5 (8.9)	57.3 (13.7)
Male	20 (60.6%)	20 (60.6%)
Female	13 (39.4%)	13 (39.4)
Race		
White	30 (90.9%)	30 (90.9%)
Black	1 (3.0%)	3 (9.1%)
Other	2 (6.1%)	0 (0.0%)
BMI	30.7 (5.0)	30.6 (6.0)
Hypertension	21 (63.6%)	21 (63.6%)
Diabetes	14 (42.4%)	15 (45.5%)
COPD	4 (12.1%)	2 (6.1%)
Smoker		
Current smoker	2 (6.1%)	2 (6.1%)
Past smoker	10 (30.3%)	11 (33.3%)
Never smoker	21 (63.6%)	20 (60.6%)
CDC wound class		
1	30 (90.9%)	32 (97.0%)
2 +	3 (9.1%)	1 (3.0%)
Hernia size		
< 5	0 (0.0%)	3 (10.3%)
> 5	25 (100%)	26 (89.7%)
Median	63.0 (18.8, 135.0)	37.5 (11.2, 96.0)
Operative characteristics		
Surgical approach		
Open	29 (87.9%)	25 (75.8%)
Laparoscopic	0 (0.0%)	1 (3.0%)
Robotic	4 (12.1%)	7 (21.2%)
Component separation performed		
None	15 (45.5%)	25 (75.8%)
Anterior/External oblique release	0 (0.0%)	4 (12.1%)
Posterior/Transverse abdominis release	18 (54.5%)	4 (12.1%)
Mesh location		
No mesh	0 (0.0%)	7 (21.2%)
Preperitoneal	21 (63.6%)	16 (48.5%)
Retromuscular	2 (6.1%)	0 (0.0%)
Bridged	2 (6.1%)	0 (0.0%)
Retrorectus	8 (24.2%)	9 (27.3%)
Retroperitoneal	0 (0.0%)	1 (3.0%)
Mesh fixation		
None	27 (81.8%)	17 (51.5%)
Sutures	6 (18.2%)	16 (48.5%)

*BMI* body mass index; *COPD* chronic obstructive pulmonary disease; *CDC* Centers for Disease Control

**Table 3:** 30-day outcomes of 1:1 matched analysis

Outcomes	Transplant (N = 33)	Non-transplant (N = 33)	*p*-value
SSO	2 (6.1%)	6 (18.2%)	0.13
SSI	1 (3.0%)	4 (12.1%)	0.16
Reoperation	1 (3.0%)	3 (9.1%)	0.30
Ileus	4 (12.1%)	1 (3.0%)	0.16
Small bowel obstruction	1 (3.0%)	0 (0.0%)	1.00
UTI	2 (6.1%)	0 (0.0%)	0.15
AKI	1 (3.0%)	1 (3.0%)	1.00
PE	0 (0.0%)	0 (0.0%)	
DVT	0 (0.0%)	0 (0.0%)	
Pneumonia	1 (3.0%)	0 (0.0%)	0.31
Respiratory failure	2 (6.1%)	0 (0.0%)	0.15
Transfer to ICU	1 (3.0%)	1 (3.0%)	0.31
Other complications	9 (27.3%)	3 (9.1%)	0.06
Readmission	3 (9.1%)	7 (21.2%)	0.17
ED visit	3 (9.1%)	6 (18.2%)	0.28
Any complications	12 (36.4%)	8 (24.2%)	0.28
Anytime hernia recurrence	4 (12.1%)	1 (3.0%)	0.16
Months for hernia recurrence	9.8 (5.4)	14.2 (N/A)	0.53

*SSO* surgical-site occurrence; *SSI* surgical-site infection; *UTI* urinary tract infection; *AKI* acute kidney insufficiency; *PE* pulmonary embolism; *DVT* deep vein thrombosis; *ICU* intensive care unit; *ED* emergency department

Thirty-day postoperative outcomes and hernia recurrence rates among transplant patients who received non-absorbable mesh (*N* = 34) vs absorbable mesh (*N* = 13) are demonstrated in Table 4. All complications were shown to be statistically insignificant. Most notably, SSO (5.9%), SSI (2.9%), reoperation (2.9%), PE (2.9%), and pneumonia (2.9%) were exclusively observed in patients with non-absorbable mesh (*p* = 1.0), while DVT was exclusively observed in patients with absorbable mesh (7.7%,* p* = 0.28). Hernias recurred at higher rates with absorbable mesh compared to non-absorbable mesh (23.1% vs 2.9%, *p* = 0.06). The median time to hernia recurrence was shorter in the absorbable group compared to the non-absorbable group (8.9 months vs 13.5 months, *p* = 0.55). Rates of any 30-day complications or adverse events were comparable between groups (35.3% non-absorbable vs. 38.5% absorbable, *p* = 1.00).

**Table 4:** 30-day outcomes by mesh type—(transplant only)

Outcomes	Non-absorbable mesh (N = 34)	Absorbable mesh (N = 13)	*p* value
SSO	2 (5.9%)	0 (0.0%)	1.00
SSI	1 (2.9%)	0 (0.0%)	1.00
Reoperation	1 (2.9%)	0 (0.0%)	1.00
Ileus	5 (14.7%)	2 (15.4%)	1.00
Small bowel obstruction	1 (2.9%)	1 (7.7%)	0.48
UTI	3 (8.8%)	1 (7.7%)	1.00
AKI	1 (2.9%)	1 (7.7%)	0.48
PE	1 (2.9%)	0 (0.0%)	1.00
DVT	0 (0.0%)	1 (7.7%)	0.28
Pneumonia	1 (2.9%)	0 (0.0%)	1.00
Respiratory failure	1 (2.9%)	2 (15.4%)	0.18
Transfer to ICU	1 (2.9%)	1 (7.7%)	0.48
Other complications	9 (26.5%)	3 (23.1%)	1.00
Readmission	3 (8.8%)	1 (7.7%)	1.00
ED visit	3 (8.8%)	1 (7.7%)	1.00
Any complications	12 (35.3%)	5 (38.5%)	1.00
Anytime hernia recurrence	1 (2.9%)	3 (23.1%)	0.06
Months for hernia recurrence	13.5 (13.5, 13.5)	8.9 (5.7, 11.6)	0.55

*SSO* surgical-site occurrence; *SSI* surgical-site infection; *UTI* urinary tract infection; *AKI* acute kidney insufficiency; *PE* pulmonary embolism; *DVT* deep vein thrombosis; *ICU* intensive care unit; *ED* emergency department

Thirty-day outcomes and hernia recurrence rates across different types of transplants are demonstrated in Table 5. Transplant types identified were limited to liver (*N* = 31), kidney (*N* = 11), heart (*N* = 3), and islet cell (*N* = 2) transplants. SSOs were reported in liver transplant recipients (3.2%) and islet cell recipients (50.0%), while no SSOs were reported in heart or kidney transplant recipients (*p* = 0.71). One case of SSI, reoperation, and PE was reported in liver transplant recipients, compared to no cases observed in the other groups (3.2%, *p* = 1). Two cases of small bowel obstruction and AKI were reported in the liver transplant cohort, compared to no cases observed in the other groups (6.5%, *p* = 1). Respiratory failure occurred in liver (3.2%) and kidney (18.2%) recipients, while pneumonia was only observed in one islet cell recipient (50.0%, *p* = 0.04). Ileus was observed in liver (12.9%), kidney (18.2%), and islet cell recipients (50.0%), with none in heart transplant recipients (*p* = 0.41). UTIs and readmissions reported similarly (6.5% liver, 9.1% kidney, 50% islet cell, *p* = 0.24). ED visits were more frequent in heart recipients (33.3%), followed by liver recipients (9.7%), with no visits in kidney or islet cell recipients (*p* = 0.32). Hernia recurrence rates were similar across liver (9.7%) and kidney (9.1%) recipients, with no recurrences observed in heart or islet cell recipients (*p* = 1). The median time to recurrence was 13.5 months in liver recipients compared to 8.9 months in kidney recipients.

**Table 5:** 30-day outcomes by transplant type

Outcomes	Liver (N = 31)	Kidney (N = 11)	Heart (N = 3)	Islet cell (N = 2)	*p* value
SSO	1 (3.2%)	0 (0.0%)	0 (0.0%)	1 (50.0%)	0.17
SSI	1 (3.2%)	0 (0.0%)	0 (0.0%)	0 (0.0%)	1.00
Reoperation	1 (3.2%)	0 (0.0%)	0 (0.0%)	0 (0.0%)	1.00
Ileus	4 (12.9%)	2 (18.2%)	0 (0.0%)	1 (50.0%)	0.41
Small bowel obstruction	2 (6.5%)	0 (0.0%)	0 (0.0%)	0 (0.0%)	1.00
UTI	2 (6.5%)	1 (9.1%)	0 (0.0%)	1 (50.0%)	0.24
AKI	2 (6.5%)	0 (0.0%)	0 (0.0%)	0 (0.0%)	1.00
PE	1 (3.2%)	0 (0.0%)	0 (0.0%)	0 (0.0%)	1.00
DVT	0 (0.0%)	1 (9.1%)	0 (0.0%)	0 (0.0%)	0.34
PNE	0 (0.0%)	0 (0.0%)	0 (0.0%)	1 (50.0%)	0.04
Respiratory failure	1 (3.2%)	2 (18.2%)	0 (0.0%)	0 (0.0%)	0.41
Transfer to ICU	0 (0.0%)	2 (18.2%)	0 (0.0%)	0 (0.0%)	0.11
Other complications	8 (25.8%)	3 (27.3%)	0 (0.0%)	1 (50.0%)	0.80
Readmission	2 (6.5%)	1 (9.1%)	0 (0.0%)	1 (50.0%)	0.24
ED visit	3 (9.7%)	0 (0.0%)	1 (33.3%)	0 (0.0%)	0.32
Any complications	11 (35.5%)	4 (36.4%)	1 (33.3%)	1 (50.0%)	1.00
Anytime hernia recurrence	3 (9.7%)	1 (9.1%)	0 (0.0%)	0 (0.0%)	1.00
Months for hernia recurrence	13.5 (8.0, 13.9)	8.9 (8.9, 8.9)	NA	NA	

*SSO* surgical-site occurrence; *SSI* surgical-site infection; *UTI* urinary tract infection; *AKI* acute kidney insufficiency; *PE* pulmonary embolism; *DVT* deep vein thrombosis; *ICU* intensive care unit; *ED* emergency department

Thirty-day postoperative outcomes and hernia recurrence between transplant patients with midline hernias (*N* = 31) and non-midline hernias (*N* = 12) are demonstrated in Table 6. Liver transplant was the most common type for both groups (67.7% midline, 58.3% non-midline), followed by kidney transplant (19.4% vs 33.3%, *p* = 0.70). No differences in SSIs were observed, with all patients included in the analysis (*p* = 1). Respiratory failure rates were low and similar (6.5% midline vs 8.3% non-midline, *p* = 1.0). SSOs (3.2%, *p* = 1), ileus (19.4%,* p* = 1), small bowel obstruction (3.2%, *p* = 1), UTI (12.9%, *p* = 1), renal failure (3.2%, *p* = 1), PE (3.2%,* p* = 1), DVT (3.2%, *p* = 1), pneumonia (3.2%, *p* = 1), readmission (9.7%, *p* = 0.55), and ED visits (9.7%, *p* = 0.55) exclusively occurred in patients with midline hernias. Hernia recurrence was only demonstrated in the non-midline hernia group (25.0% vs 0.0%, *p* = 0.02). One patient had a recurrence of both midline and non-midline located hernia components and was excluded from the analysis. The median time to hernia recurrence in the non-midline group was 8.9 months.

**Table 6:** 30-day outcomes by hernia location (transplant only)

Outcomes	Midline (N = 31)	Non-midline (N = 12)	*p* value
Previous transplant			
1-Liver	21 (67.7%)	7 (58.3%)	
2-Kidney	6 (19.4%)	4 (33.3%)	
3-Heart	2 (6.5%)	1 (8.3%)	
5-Islet cell	2 (6.5%)	0 (0.0%)	
SSO	1 (3.2%)	0 (0.0%)	1.00
SSI	1 (3.2%)	0 (0.0%)	1.00
reoperation	0 (0.0%)	0 (0.0%)	
Ileus	6 (19.4%)	0 (0.0%)	0.16
Small bowel obstruction	1 (3.2%)	0 (0.0%)	1.00
UTI	4 (12.9%)	0 (0.0%)	0.56
AKI	1 (3.2%)	0 (0.0%)	1.00
PE	1 (3.2%)	0 (0.0%)	1.00
DVT	1 (3.2%)	0 (0.0%)	1.00
PNE	1 (3.2%)	0 (0.0%)	1.00
Respiratory failure	2 (6.5%)	1 (8.3%)	1.00
Transfer to ICU	1 (3.2%)	1 (8.3%)	0.49
Other complications	6 (19.4%)	4 (33.3%)	0.43
Readmission	3 (9.7%)	0 (0.0%)	0.55
ED visit	3 (9.7%)	0 (0.0%)	0.55
Any complications	11 (35.5%)	4 (33.3%)	1.00
Anytime hernia recurrence	0 (0.0%)	3 (25.0%)	0.02
Months for hernia recurrence	NA	8.9 (5.7, 11.6)	

*SSO* surgical-site occurrence; *SSI* surgical-site infection; *UTI* urinary tract infection; *AKI* acute kidney insufficiency; *PE* pulmonary embolism; *DVT* deep vein thrombosis; *ICU* intensive care unit; *ED* emergency department

Outcomes for immunosuppressant types, tacrolimus (N = 29), sirolimus (N = 5), and other immunosuppressants (N = 13), used in transplant patients are outlined in Table 7. SSIs and reoperation were exclusively reported in the sirolimus group (20.0%, *p* = 0.11). PE (3.4%), DVT (3.4%), respiratory failure (10.3%), and 30-day ICU transfer (6.9%) were exclusively reported in the Tacrolimus group. Higher rates of SSOs were seen in patients taking sirolimus (20.0%) and other medications (7.7%) compared to those taking tacrolimus (p = 0.07). No difference was observed across the cohorts when evaluating ileus or UTI rates (p = 1.0). AKI occurred in one patient each in the tacrolimus (3.4%) and sirolimus (20.0%) cohorts (p = 0.28). Small bowel obstruction was observed in the tacrolimus (3.4%) and sirolimus (20.0%) groups (*p* = 0.28). Readmissions (6.9% tacrolimus, 20% sirolimus, 7.7% other, *p* = 0.54) and ED visits (10.3% tacrolimus, 20% sirolimus, 0.0% other, *p* = 0.66) were similar. Other complications, such as hyperglycemia or anemia, occurred in the tacrolimus (27.6%), sirolimus (40.0%), and other (15.4%) without an observed difference (*p* = 0.46). Rates of any complications or adverse events were similar across groups (*p* = 0.54).

**Table 7:** 30-day outcomes by immunosuppressant (transplant only)

Outcomes	Tacrolimus (N = 29)	Sirolimus (N = 5)	Other (N = 13)	*p* value
Previous transplant				0.50
1-Liver	20 (69.0%)	4 (80.0%)	7 (53.8%)	
2-Kidney	7 (24.1%)	1 (20.0%)	3 (23.1%)	
3-Heart	2 (6.9%)	0 (0.0%)	1 (7.7%)	
5-Islet cell	0 (0.0%)	0 (0.0%)	2 (15.4%)	
SSO	0 (0.0%)	1 (20.0%)	1 (7.7%)	0.07
SSI	0 (0.0%)	1 (20.0%)	0 (0.0%)	0.11
Reoperation	0 (0.0%)	1 (20.0%)	0 (0.0%)	0.11
Ileus	4 (13.8%)	1 (20.0%)	2 (15.4%)	1.00
Small bowel obstruction	1 (3.4%)	1 (20.0%)	0 (0.0%)	0.28
UTI	3 (10.3%)	0 (0.0%)	1 (7.7%)	1.00
AKI	1 (3.4%)	1 (20.0%)	0 (0.0%)	0.28
PE	1 (3.4%)	0 (0.0%)	0 (0.0%)	1.00
DVT	1 (3.4%)	0 (0.0%)	0 (0.0%)	1.00
PNE	0 (0.0%)	0 (0.0%)	1 (7.7%)	0.38
Respiratory failure	3 (10.3%)	0 (0.0%)	0 (0.0%)	0.67
Transfer to ICU	2 (6.9%)	0 (0.0%)	0 (0.0%)	1.00
Other complications	8 (27.6%)	2 (40.0%)	2 (15.4%)	0.55
Readmission	2 (6.9%)	1 (20.0%)	1 (7.7%)	0.56
ED visit	3 (10.3%)	1 (20.0%)	0 (0.0%)	0.28
Any complications	12 (41.4%)	2 (40.0%)	3 (23.1%)	0.54
Anytime hernia recurrence	0 (0.0%)	2 (40.0%)	2 (15.4%)	0.01
Months for hernia recurrence	NA	11.2 (10.1, 12.4)	8.4 (5.5, 11.4)	

*SSO* surgical-site occurrence; *SSI* surgical-site infection; *UTI* urinary tract infection; *AKI* acute kidney insufficiency; *PE* pulmonary embolism; *DVT* deep vein thrombosis; *ICU* intensive care unit; *ED* emergency department

Hernia recurrence showed variation across groups, occurring in 40.0% of the sirolimus group and 15.4% of the other immunosuppressants group but none in the Tacrolimus group (*p* = 0.01). The median time to recurrence was 11.2 months for patients taking sirolimus and 8.4 months for patients taking other immunosuppressants.

## Discussion

Transplant numbers and the use of immunosuppressants continue to rise, due to large advancements in the care of the transplant patient. There continues to be an insufficient amount of data on how to care for these patients and optimize outcomes in VHR. Our study elucidates outcomes in patients facing VHR with prior transplants versus case-matched patients with no prior transplant. We were able to find that the immunosuppression does not seem to affect hernia recurrence. However, the type of immunosuppression especially sirolimus may worsen outcomes. This data will help surgeons make recommendations when planning the repair of these complex patients.

The first interesting finding in this study is that the matched analysis did not demonstrate a significant difference in thirty-day outcomes. Our study found no difference between SSOs and SSIs within 30 days postoperative despite previous studies evaluating transplant recipients have shown increased rates of SSIs and SSOs. The increased rates of SSO and SSI in previous studies are thought to be secondary to immunosuppression and poor wound healing in this demographic [16–18]. Solid organ transplant recipients undergoing VHR have demonstrated increased rates of wound complications, notably seroma formation, that is not seen in our study [5, 19]. Our findings of equivalency are likely multifactorial and could be attributed to advances in hernia surgery, dedicated multidisciplinary care, or an evolving standardized pathway. We cannot discount the small sample size of the study, however, some additional studies have shown equivalency regarding both SSO and SSI rates [20, 21]. Hernias did occur at a higher rate in the transplant cohort compared to the nontransplant cohort based on our data. Previously this finding was postulated to be secondary to wound healing defects in immunosuppressed patients; however, our data did not demonstrate increased wound complications in the 30-day postoperative period. One explanation for this based on our data is that two of the recurrences in our transplant cohort were after a bridged repair in which the fascia was unable to be completely closed. This leads to an increased rate of hernia recurrence. Though, Lee et al. also showed no increased risk of postoperative SSI, yet an increased hernia recurrence rate was seen in an immunosuppressed cohort [22]. This finding, however, although interesting, was not significant in our study likely secondary to sample size and is less significant if bridged repairs are excluded. These studies and the data of the current study suggest it is safe for patients with previous transplant surgery to undergo ventral hernia repair without an increased risk for SSO and SSI and recurrence rates may need further evaluation to demonstrate any increased risk.

The second important finding in this study is looking at the type of mesh used in the patient’s repair. All transplant patients in our study received mesh implantation at the time of VHR. Overall, while most complications were similar between the two groups, the most notable difference was the higher rate of hernia recurrence in the absorbable mesh group (23.1%) compared to the non-absorbable mesh group (2.9%) (p = 0.06). The shorter median time to recurrence in the absorbable mesh group (8.9 months vs. 13.5 months) and increased hernia recurrence with absorbable mesh are likely due to mesh absorption and repair type as two of these recurrences were after bridged repairs. This aligns with recent literature suggesting that non-absorbable meshes may provide better long-term outcomes in hernia repairs compared to absorbable alternatives. Rodriguez-Quintero et al. demonstrated that permanent synthetic mesh had a lower recurrence rate (23%) compared to absorbable synthetic mesh (40%) at 1 year in contaminated fields [21]. Additionally, it shows that tension-free fascial closure is paramount to preventing recurrence. However, selection bias can play a role in this finding as patients at higher risk of complications are more likely to receive absorbable mesh.

The third interesting finding is that our related sub-set analysis evaluating the location of ventral hernia, either midline or non-midline, shows similar 30-day adverse outcome results between the two cohorts. However, three of the hernias that recurred with a single hernia component were non-midline hernias (p = 0.02). The fourth hernia recurrence had multiple hernias present that included both a non-midline and a midline component. These results indicate that hernia location, whether midline or non-midline, played a role in our study. There is little research comparing midline and non-midline hernia location and their postoperative outcomes. Non-midline hernias may be at an increased risk due to anatomical location near bony structures, increased tension, technical challenges of achieving adequate mesh overlap, and increased risk for neurovascular denervation of the abdominal wall. Increased lateral intra-abdominal pressure may also be at play when considering the cause of this finding. Given that two of these non-midline recurrences occurred following a bridged repair, it suggests that the complexity of these cases may necessitate bridging techniques due to the difficulty in achieving a tension-free fascial closure. Careful consideration of surgical technique and dissection must be taken, and non-midline located ventral hernias require a more complex approach or different mesh selection process to improve durability and reduce recurrence.

The fourth interesting finding in this study was the analysis of outcomes in transplant patients undergoing ventral hernia repair based on immunosuppressant medications. There is a gap in the literature directly comparing outcomes with different immunosuppressants in the perioperative period after VHR. There were notable differences in hernia recurrence rates across the cohorts in our data. Tacrolimus-treated patients experienced no hernia recurrences, while 40% of Sirolimus patients and 15.4% of those on other immunosuppressants had recurrences (*p* = 0.01). Both recurrences in the “other” cohort were bridged repairs, and when excluded would demonstrate improved rates for this cohort. However, the recurrences in patients that were on sirolimus occurred in patients where complete fascial closure was achieved, which makes sirolimus treated patients undergoing VHR of particular interest. Han et al. showed a recurrence rate of 4.5% in postoperative VHR patients taking tacrolimus with a larger sample size; however, this was in a population of patients undergoing laparoscopic intervention only [23]. Additionally, Weiss et al. showed that sirolimus use was an independent risk factor for hernia recurrence in a cohort of liver transplant patients undergoing VHR [24]. Sirolimus has been associated with a higher incidence of wound-healing complications, which can contribute to increased rates of hernia recurrence. Dean et al. conducted a prospective, randomized trial comparing sirolimus and tacrolimus in renal allograft recipients and found that the incidence of wound complications was 47% in the sirolimus group compared to 8% in the tacrolimus group [25]. The mTOR inhibitor, sirolimus, is often given to patients who have failed tacrolimus, with an oncologic history or impaired kidney function which are not controlled for in our data, introducing possible confounders. Another variable of interest lies in postoperative weight gain, as it is well established that high body mass index is correlated with hernia recurrence [26–29]. A link specific to postoperative weight gain and hernia recurrence has not been specifically defined in literature. Nonetheless, immunosuppressive agents have been shown to lead to increased weight gain compared to patients that are not on an immunosuppressive regimen [30–32]. The proposed mechanism specific to calcineurin and mTOR inhibitors involves decreased insulin-stimulated glucose uptake, primarily through downregulation of the GLUT4 receptor [30–32]. Further studies evaluating the role for weight gain in immunosuppressed patients who experience hernia recurrence and prospective studies directly comparing outcomes in patients on sirolimus and tacrolimus could further clarify these results. However, given these findings, there should be additional consideration of the type of immunosuppressant when managing transplant patients for hernia repair, particularly regarding long-term outcomes like hernia recurrence. Our current pathway converts patients from sirolimus to tacrolimus for the preoperative period when feasible.

Some limitations exist in our study that should be considered during interpretation. Due to the retrospective study design, biases inherent to this design, such as selection bias, could be present. Additionally, the sample size for the transplant cohort, particularly for sub-set analyses involving transplant type and immunosuppressant medications, was relatively small. This limited statistical power could have hindered the detection of significant differences in outcomes, especially for less common complications, and increases the risk of Type II error. Additionally, the follow-up time in our study of 13 months could underestimate the true recurrence numbers. A multi-center approach to allow for increased patient numbers, and thus increased power, would be ideal for additional analyses. Future studies should focus on prospective cohorts and randomized controlled trials to further evaluate these critical variables in transplant patients undergoing VHR.

In conclusion, our study shows that operating on transplant patients for ventral hernias is safe when compared to a similar cohort of non-transplant patients. All 30-day outcomes matched those of patients outside the study cohort, and this was regardless of the type of mesh. The largest differences we found were that the location of the hernia defect and repair may impact outcomes as well as the type of immune suppression the patient is taking. Heightened awareness should be used in patients undergoing non-midline hernias, and temporary use of an immunosuppression regimen including tacrolimus versus sirolimus should be considered.
